# Gastrointestinal Symptoms and Dopamine Transporter Asymmetry in Early Parkinson's Disease

**DOI:** 10.1002/mds.28986

**Published:** 2022-03-11

**Authors:** Kirsi Murtomäki, Tuomas Mertsalmi, Elina Jaakkola, Elina Mäkinen, Reeta Levo, Tanja Nojonen, Mikael Eklund, Simo Nuuttila, Kari Lindholm, Eero Pekkonen, Juho Joutsa, Tommi Noponen, Toni Ihalainen, Valtteri Kaasinen, Filip Scheperjans

**Affiliations:** ^1^ Department of Neurology Helsinki University Hospital, and Clinicum, University of Helsinki Helsinki Finland; ^2^ Clinical Neurosciences University of Turku, and Neurocenter, Turku University Hospital Turku Finland; ^3^ Turku PET Centre Turku University Hospital Turku Finland; ^4^ Turku Brain and Mind Center University of Turku Turku Finland; ^5^ Department of Clinical Physiology and Nuclear Medicine, Department of Medical Physics Turku University Hospital Turku Finland; ^6^ HUS Medical Imaging Center Clinical Physiology and Nuclear Medicine, University of Helsinki and Helsinki University Hospital Helsinki Finland

**Keywords:** Parkinson's diseasegastrointestinal symptomsdopamine transporter

## Abstract

**Background:**

The neurophysiological correlates of gastrointestinal symptoms (GISs) in Parkinson's disease (PD) are not well understood. It has been proposed that in patients with a gastrointestinal origin of PD dopaminergic neurodegeneration would be more symmetric.

**Objectives:**

The aim is to assess the associations between GISs and asymmetry of nigrostriatal dopaminergic neurodegeneration in PD.

**Methods:**

Ninety PD patients were assessed using motor and GIS scales and ^123^I‐FP‐CIT SPECT. We calculated the asymmetry index and the predominant side of motor symptoms and dopamine transporter (DAT) imaging defect and assessed their association with GISs.

**Results:**

There were no significant differences in GISs between symmetric and asymmetric dopaminergic defect. Left predominant defect was related to more GIS and higher constipation scores.

**Conclusions:**

GISs were associated with left predominant reduction in putaminal DAT binding but not asymmetry per se. It remains open whether left‐sided DAT deficit is related to more pronounced GI involvement or symptom perception in PD. © 2022 The Authors. *Movement Disorders* published by Wiley Periodicals LLC on behalf of International Parkinson Movement Disorder Society.

Parkinson's disease (PD) is a progressive neurodegenerative disorder characterized by motor and nonmotor manifestations.^1^ Typically, motor symptoms emerge unilaterally or asymmetrically.^2^ A considerable subgroup of patients suffer from gastrointestinal symptoms (GISs) such as functional constipation (FC) or irritable bowel syndrome (IBS),^3^, ^4^, ^5^ and autopsy studies have shown α‐synuclein pathology throughout the spinal cord and peripheral nervous systems.^6^

Recently, an α‐synuclein origin site and connectome (SOC) model has been suggested, dividing PD into brain‐first and body‐first subtypes.^7^ In the brain‐first subtype, the α‐synuclein pathology spreads mainly unilaterally, whereas in the body‐first subtype, pathology is more symmetric due to the bilateral spreading of α‐synuclein via the vagus nerve.

Dopamine transporter (DAT) imaging with single‐photon emission computerized tomography (SPECT) is an in vivo biomarker of nigrostriatal neuron loss. The correlation between the motor symptoms and the striatal DAT binding is well recognized; however, the relation between nonmotor symptoms and DAT binding has been investigated in only a few studies.^8^, ^9^ In two studies on the Parkinson's Progressive Markers Initiative cohort, nonmotor symptoms have been associated with reduced striatal DAT binding.^10^, ^11^

We hypothesized that in early‐stage PD patients, less asymmetric DAT deficit is associated with more GISs.

## Patients and Methods

Ninety PD patients were imaged using ^123^I‐FP‐CIT SPECT because of parkinsonism or tremor in 2015–2019 at Helsinki and Turku University Hospitals (NMDAT study, ClinicalTrials.gov identifier NCT02650843). See the supplement for inclusion and exclusion criteria.

The study was approved by the Ethics Committee of the Turku University Hospital and was conducted according to the principles of the Declaration of Helsinki. All participants provided written informed consent.

### SPECT Imaging and Data Analysis

See the supplement for technical details of SPECT imaging. Specific DAT binding was calculated from striatal subregions (caudate, anterior putamen, and posterior putamen) using region‐to‐occipital cortex ratio with BRASS software (Table S3).^12^

The limit for an abnormal scan was more than two standard deviations below the reference mean in any of the six analyzed regions.

Mean putamen DAT binding was calculated for both sides as mean putamen = (anterior putamen SBR + posterior putamen SBR)/2.

Mean putamen asymmetry index (AI) in DAT uptake was then calculated as AI = (putamenhighest − putamenlowest)/(putamenhighest + putamenlowest). AI > 0.05 was considered as significantly asymmetric reduction in DAT binding.^13^

### Clinical Features

Assessments are summarized in Table 1. The MDS‐UPDRS, Part III, total score and subscores for tremor, bradykinesia, and rigidity were separately calculated for both sides^13^ (see Appendix S1). AI was calculated for all these lateralized scores.^13^ Significant asymmetry was defined as AI > 0.3.

**TABLE 1:** Basic characteristics of PD patients

Characteristics	PD
Number of patients	90
Age, mean ± SD	65.29 ± 9.97
Sex: male, n (%)	46 (51.1)
Handedness: right, n (%)	85 (94.4)
MMSE,^14^ mean ± SD	27.51 ± 1.86
Motor symptoms in months, mean ± SD	28.92 ± 31.09
Hoehn and Yahr (H&Y) score, mean ± SD	1.99 ± 0.71 H&Y 1, N (%)21 (23.3)
H&Y 2, N (%)	51 (56.7)
H&Y 3, N (%)	16 (17.8)
H&Y 4, N (%)	2 (2.2)
MDS‐UPDRS III total score,^15^ mean ± SD	34.72 ± 13.57
MDS‐UPDRS III tremor score,^13^ mean ± SD	4.60 ± 3.27
MDS‐UPDRS III bradykinesia‐rigidity score,^13^ mean ± SD	19.08 ± 9.39
NMSS total score,^16^ mean ± SD	41.68 ± 31.72
Dream enactment (possible RBD),^17^ N (%)	24 (26.7)
Functional gastrointestinal disorders, N (%)	32 (35.6)
IBS, n (%)	17 (18.9)
Functional dyspepsia, n (%)	14 (15.5)
Functional constipation, n (%)	4 (4.4)
Functional bloating, n (%)	3 (3.3)
Functional diarrhea, n (%)	1 (1.1)
Rome III constipation subscore,^18^ mean ± SD	5.96 ± 5.13
Wexner total score,^19^ mean ± SD	5.04 ± 4.05
CSI total score,^20^ mean ± SD	15.20 ± 11.30
NMSS constipation score, mean ± SD	1.66 ± 3.33
Mean putamen absolute value, mean ± SD	0.14 ± 0.10
Patients with dopaminergic medication, N (%)	25 (28.1)
Mean levodopa equivalent daily dose, mean ± SD (range)	82.27 ± 155.36 (0–600)

Wexner and CSI are constipation questionnaires. Rome III assesses functional gastrointestinal disorders.

Abbreviations: PD, Parkinson's disease; SD, standard deviation; MMSE, Mini‐Mental State Examination; N, number of patients; MDS‐UPDRS III, MDS Unified Parkinson's Disease Rating Scale, Part III; NMSS, Non‐Motor Symptoms Scale; IBS, irritable bowel syndrome; CSI, Constipation Severity Instrument.

We used Rome III criteria for the identification of functional gastrointestinal disorders (FGIDs): IBS, FC, functional diarrhea, functional dyspepsia, and functional bloating.

### Imaging Asymmetry and Clinical Symptoms

Based on the motor symptoms' AIs, 50 (55.6%) patients had predominantly symmetric, 20 (22.2%) predominantly right‐sided, and 20 (22.2%) predominantly left‐sided motor symptoms at the time of imaging. In many of the previous studies, the laterality has been analyzed based on motor symptoms, but we also utilized dopamine imaging data. The motor symptoms AI correlated with the imaging AI best for mean putamen (Figure S1).

### Statistical Analyses

All the analyses were performed using IBM SPSS Statistics version 25 (see Appendix S1 for details).

## Results

### GISs in PD Patients

Basic cohort characteristics are summarized in Table 1 and Table S1. Thirty‐two patients (35.6%) fulfilled the criteria for at least one FGID.

### Asymmetry Indexes and GISs

In mean putamen, 72 (80.0%) patients had asymmetric and 18 (20.0%) symmetric reduction in DAT binding. The majority of the patients (90%) reported asymmetric and 10% symmetric onset of motor symptoms. Degree of asymmetry of mean putamen DAT binding showed no statistically significant association with any clinical measure (Table 2).

**TABLE 2:** . PD patients divided into asymmetric and symmetric groups based on mean putamen asymmetry index

	Mean putamen imaging deficiency		*P‐*value
	Asymmetric (N = 72)	Symmetric (N = 18)	
Patients, N (%)	72 (80.0)	18 (20.0)
Age, mean ± SD	64.62 ± 9.94	68.78 ± 9.54	0.082
Sex: male, N (%)	35 (48.6)	11 (61.1)	0.34
MMSE, mean ± SD	27.39 ± 1.92	28.00 ± 1.57	0.26
Motor symptoms in months, mean ± SD	27.64 ± 25.67	34.06 ± 47.69	0.60
Nonmotor symptoms in months, mean ± SD	136.83 ± 173.18	102.33 ± 182.06	0.071
Hoehn & Yahr, mean ± SD	1.94 ± 0.75	2.17 ± 0.51	0.16
MDS‐UPDRS III total score, mean ± SD	33.54 ± 13.46	39.44 ± 13.34	0.099
MDS‐UPDRS III tremor score, mean ± SD	4.42 ± 3.11	5.33 ± 3.85	0.41
MDS‐UPDRS III bradykinesia‐rigidity score, mean ± SD	18.42 ± 9.36	21.72 ± 9.30	0.17
NMSS total score, mean ± SD	44.13 ± 33.57	31.89 ± 20.82	0.22
Dream enactment (possible RBD), N (%)	18 (25.0)	6 (33.3)	0.70
Functional gastrointestinal disorders, N (%)	28 (38.9)	4 (22.2)	0.19
IBS, N (%)	16 (22.2)	1 (5.6)	0.11
Dyspepsia, N (%)	10 (13.9)	2 (22.2)	0.38
Rome III constipation subscore, mean ± SD	6.13 ± 5.55	5.28 ± 2.97	0.94
Wexner sum, mean ± SD (N = 88)	5.38 ± 4.31	3.68 ± 2.46	0.29
CSI total score, mean ± SD (N = 89)	15.13 ± 11.80	15.47 ± 9.33	0.62
NMSS constipation score, mean ± SD	1.61 ± 0.39	1.83 ± 0.81	0.47

Wexner and CSI are constipation questionnaires. Rome III assesses functional gastrointestinal disorders.

Abbreviations: PD, Parkinson's disease; SD, standard deviation; N, number of patients; MMSE, Mini‐Mental State Examination; MDS‐UPDRS III, MDS Unified Parkinson's Disease Rating Scale, Part III; NMSS, Non‐Motor Symptoms Scale; IBS, irritable bowel syndrome; CSI, Constipation Severity Instrument.

When compared to patients with symmetric or right‐predominant reduction in mean putamen DAT binding, FGIDs (in particular IBS) were significantly more common and Wexner constipation scores higher in patients with left‐predominant reduction (Table S2; Figure S2).

## Discussion

The aim of this study was to elucidate the association between nigrostriatal DAT binding asymmetry and GISs in PD. Falsifying our initial hypothesis, more symmetrical binding was not associated with increased GISs.^7^ Instead, FGIDs and symptoms of constipation were associated with left‐predominant putaminal reduction in DAT binding. Our results suggest that not asymmetry per se but rather left‐predominant defect is associated with reported GISs in PD.

The proportion of PD patients fulfilling the criteria for IBS (18.9%, n = 17) observed in this study is in line with previous findings in Finnish (24.3%) and Japanese (27.1%) populations.^5^, ^21^ On the contrary, the prevalence of FC (4.4%) was quite low compared to earlier observations in the Finnish and Japanese cohorts (12.2% and 17.1%, respectively). A previous Korean study reported higher prevalence of FC (74.3%) and lower prevalence of IBS (2.9%) in PD, whereas the prevalence of functional dyspepsia (14.3%) was comparable to our results.^22^

Previous studies have suggested a relation between the laterality of dopaminergic neurodegeneration and nonmotor symptoms in PD. In contrast to our study, the study by Deursen and colleagues showed that autonomic symptoms were related to lower ^123^I‐FP‐CIT binding ratios in the right caudate nucleus and mainly driven by gastrointestinal [Scales for Outcomes in Parkinson's Disease—Autonomic (SCOPA‐AUT) score] and cardiovascular dysfunction.^23^ Differences between the results could be explained by the patient population. Deursen's study had a larger sample size (310 vs. 90), the mean levodopa equivalent daily dose was higher (136.70 vs. 82.27 mg), and the disease duration was longer (3.68 vs. 2.5 years) compared to our study. The SCOPA‐AUT has only seven questions on GISs, whereas the Rome III has 27, assessing GISs more widely. Further, in Miyamoto's study, right‐handed idiopathic rapid eye movement sleep behavior disorder (iRBD) patients had faster degeneration in the left hemisphere.^24^ A recent study found a relation between left‐hemispheric dopaminergic denervation and cognitive impairment in PD.^25^ Further, previous studies have suggested laterality of PD motor symptoms to be associated with nonmotor symptoms with mixed results.

Psychosis has been associated with right‐predominant motor symptoms.^9^ On the contrary, PD patients with right‐predominant motor dysfunction appear to exhibit greater cognitive impairment.^26^, ^27^ Thus, lateralization may influence nonmotor symptoms.

The SOC model posits that α‐synuclein pathology is the core feature in PD pathogenesis. The anatomical location of the first pathogenic α‐synuclein is important, and the neural connectome also plays a crucial role in determining how pathological α‐synuclein propagates through the nervous system.^7^ If the α‐synuclein pathology starts from the gut and slowly propagates caudo‐rostrally through the vagal nerves, which do not show strong lateralization, to the dorsal motor nuclei of vagus and then bilaterally to the brainstem, we would expect predominantly symmetric DAT binding defect. A recent article by Van Den Berge and Ulusoy discussed that animal models also support the SOC model, with more symmetric α‐synuclein spreading after peripheral initiation of pathology as compared to stronger asymmetry after intracerebral initiation.^28^ Environmental toxins in both animals and humans mostly cause bilateral parkinsonism.^27^ Recent pathological evidence supports the existence of two patterns of α‐synuclein pathology with caudo‐rostral or amygdala‐based progression.^29^ A cortical pathogenic theory postulates the existence of top‐down corticostriatal processes that could drive asymmetric retrograde nigrostriatal degeneration.^30^ Therefore, multiple mechanisms may govern the degree of asymmetry of PD pathology and symptoms.

Although our results did not confirm a relation between subjective GISs and stronger symmetry of dopaminergic neurodegeneration, our results do not necessarily argue against the SOC model. The GI tract is regulated by the local nervous system and the vagal nerve. Furthermore, the information spreads through the vagal nerve to the striatum but also wider in the central nervous system. Consequently, the correlation between subjective GISs and objective dysfunction has been far from optimal.^31^, ^32^ Also, abnormal gut motility, visceral hypersensitivity, inflammation, or altered bacterial flora may influence the GISs. In addition, female patients tend to have more GISs than male patients, but there were no significant differences in our study.

Overall, patients' symptom experience is a combination of different mechanisms, and it cannot be directly inferred that patients with more severe subjective GISs have more gut pathology.

The main strength of this study is that the laterality of PD pathology was assessed using ^123^I‐FP‐CIT SPECT and not only by the distribution of motor symptoms. However, the definition of symmetrically reduced dopaminergic activity is artificial. We chose a previously used definition of AI value between −0.05 and 0.05, and it is possible that other cutoff values would produce different results.^13^

Another strength of this study is that the diagnosis of PD was confirmed using the ^123^I‐FP‐CIT SPECT, and patients were followed to further minimize the probability of false PD diagnosis (the mean follow‐up time being 30 months).

Given that patients were sent to imaging for diagnosis, most of them had early‐stage symptoms without long use of dopaminergic medication or were drug‐naive patients. Nevertheless, the average time since motor symptom onset in our cohort was 29 months and, nonsignificantly, longer in symmetric (34 months) than in asymmetric (27.6 months) patients. This is longer than in previous imaging studies of RBD‐positive (17 months) and RBD‐negative (21.5 and 24 months, respectively) PD patients, and we cannot exclude that diminishing of asymmetry during disease progression influenced our results.^33^, ^34^ It is also possible that in our cohort there was clinical uncertainty about PD diagnosis, prompting referral to ^123^I‐FP‐CIT SPECT imaging to confirm the diagnosis and leading to selection bias. These PD patients might thus differ from the general PD population in terms of the underlying etiologic factors, and results cannot necessarily be generalized to the entire PD population.

One limitation in our study is that the GISs are based only on questionnaires. No objective functional gastrointestinal investigations were carried out.^31^ However, in a study on PD patients with constipation and patients with FC, the patients had severe functional dysmotility of the colon and rectum, based on transit test and manometry,^32^ supporting using the Rome questionnaire.

In conclusion, GIS burden was not associated with the degree of symmetry of DAT defect but rather with left‐sided DAT decrease. Further studies using objective biomarkers to assess GI pathology and dysfunction are needed to establish whether left‐sided DAT deficit is related to more pronounced GI involvement or rather symptom perception in PD.

## Full financial disclosures for the previous 12 months

K.M. received grants from the Finnish Parkinson Foundation, the Hospital District of Helsinki and Uusimaa, and the Maire Taponen Foundation; T.M. received grants from the Finnish Parkinson Foundation, Emil Aaltonen's Foundation, and the Finnish‐Norwegian Medical Foundation, Employment Helsinki University Hospital, Neuroinnovation Oy; E.J. received grants from the Finnish Parkinson Foundation; E.M.: none; R.L.: none; T.N.: none; M.E. received grants from Turku University Hospital (ERVA funds), the Finnish Parkinson Foundation, and The Finnish Medical Foundation; S.N. holds stock ownership in medically related fields; K.L.: none; E.P. received the Finnish Government Research grant; lecturer honorarium from Abbott, AbbVie, and NordicInfucare; and consulting fees from Boston Scientific, NordicInfu Care AB, and AbbVie. PI in Finland: International Adroit‐study (Abbott DBS Registry of Outcomes for Indications over Time). 2021–: organized by Abbott; J.J. received a lecturer honorarium from Lundbeck; T.N. received grants from the Turku University Hospital (VTR funds); T.I.: none; V.K. received grants from the Päivikki and Sakari Sohlberg Foundation; serves on the advisory boards of AbbVie and Nordic Infucare AB; and received honoraria from AbbVie, Nordic Infucare AB, Orion Pharma, and Adamant Health; F.S. has received grants from the Academy of Finland, the Hospital District of Helsinki and Uusimaa, OLVI‐Foundation, Konung Gustaf V:s och Drottning Victorias Frimurarestiftelse, The Wilhelm and Else Stockmann Foundation, The Emil Aaltonen Foundation, the Yrjö Jahnsson Foundation, Renishaw, and received honoraria from AbbVie, Orion, GE Healthcare, Merck, Teva, Bristol Myers Squibb, Sanofi, and Biogen.

## Author Roles

K.M.: execution, analysis, writing, and editing of the final version of the manuscript; T.M.: execution, analysis, writing, and editing of the final version of the manuscript; E.J.: execution and editing of the final version of the manuscript; E.M.: execution and editing of the final version of the manuscript; R.L.: execution and editing of the final version of the manuscript; T.N.: execution and editing of the final version of the manuscript; M.E.: execution and editing of the final version of the manuscript; S.N.: execution and editing of the final version of the manuscript; K.L.: execution and editing of the final version of the manuscript; E.P.: editing of the final version of the manuscript; J.J.: design, execution, and editing of the final version of the manuscript; T.N.: execution and editing of the final version of the manuscript; T.I.: analysis and editing of the final version of the manuscript; V.K.: design, execution, and editing of the final version of the manuscript; F.S.: design, execution, and editing of the final version of the manuscript.

## Supporting information


**Figure S1.** Scatterplot of motor symptoms AI and mean putamen AI correlation. The X‐axis shows mean putamen AI. The Y‐axis shows motor symptoms AI. Spearman correlation ρ = 0.745 (*p* < 0.001) Negative motor symptoms AI (based on MDS‐UPDRS III) means left sided motor symptoms and negative mean putamen AI means right sided defect. AI = asymmetry index.

**Figure S2.** Correlation between mean putamen AI and Wexner sum score. Scatterplot against asymmetry index for Wexner score. Spearman correlation ρ = 0.20 (*p* = 0.067). Also 95% confidence interval lines shown. Negative mean putamen AI means right sided defect. Abbreviations: AI = asymmetry index.

**Table S1.** Patients with FGIDs and without FGIDs, basic information.

**Table S2.** PD patients divided into groups based on the mean putamen asymmetry index.

**Table S3.** SBR valuesand Z‐scorein PD patients in putamen and nucleus caudatus.

**Appendix S1**. Supporting Information.

## Data Availability

Data available on request due to privacy/ethical restrictions
