# Supplementary figures and images for: Gastrointestinal Symptoms and Dopamine Transporter Asymmetry in Early Parkinson's Disease
Source: Mov Disord. 2022 Mar 11;37(6):1284–9. doi: 10.1002/mds.28986 (PMC9314058; doi:10.1002/mds.28986)

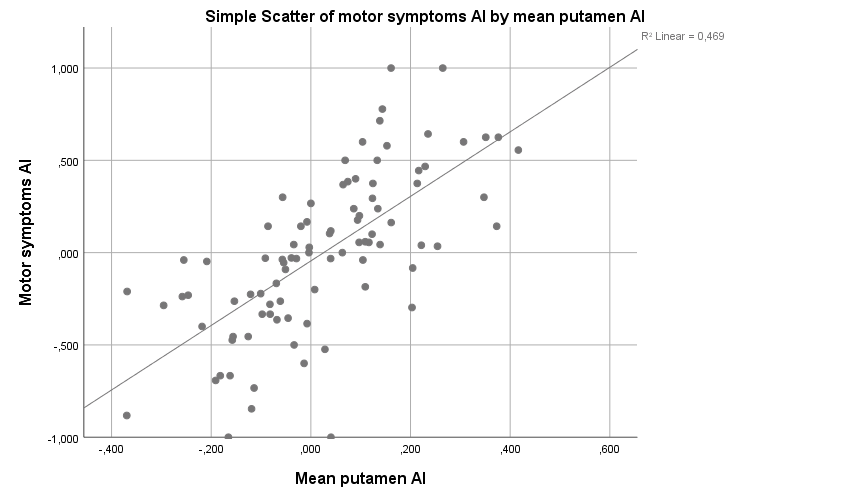

Supplement: Supplementary file 1 — Figure S1. Scatterplot of motor symptoms AI and mean putamen AI correlation. The X‐axis shows mean putamen AI. The Y‐axis shows motor symptoms AI. Spearman correlation ρ = 0.745 (p < 0.001) Negative motor symptoms AI (based on MDS‐UPDRS III) means left sided motor symptoms and negative mean putamen AI means right sided defect. AI = asymmetry index. [file MDS-37-1284-s006.tif]

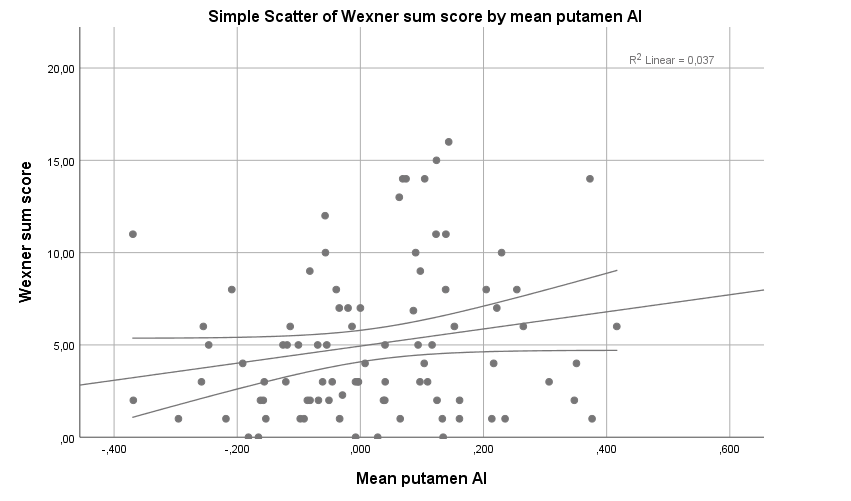

Supplement: Supplementary file 2 — Figure S2. Correlation between mean putamen AI and Wexner sum score. Scatterplot against asymmetry index for Wexner score. Spearman correlation ρ = 0.20 (p = 0.067). Also 95% confidence interval lines shown. Negative mean putamen AI means right sided defect. Abbreviations: AI = asymmetry index. [file MDS-37-1284-s004.tif]
