# Supplementary material for: Gastrointestinal Symptoms and Dopamine Transporter Asymmetry in Early Parkinson's Disease
Source: Mov Disord. 2022 Mar 11;37(6):1284–9. doi: 10.1002/mds.28986 (PMC9314058; doi:10.1002/mds.28986)
Supplement: Supplementary file 3 — Table S1. Patients with FGIDs and without FGIDs, basic information. [file MDS-37-1284-s002.pdf]

Table S1 Patients with FGIDs and without FGIDs, basic information.

	FGIDs + (N=32)	FGIDs - (N=58)	p value
Age, mean ± SD	67.69 ± 6.90	63.97 ± 11.14	0.22
Sex; Male, N(%)	12 (37.5)	34 (58.6)	0.055
MMSE, mean ± SD	27.00 ± 2.06	27.79 ± 1.69	0.087
Motor symptoms in months, mean ± SD	23.63 ± 24.26	31.84 ± 34.13	0.16
Hoehn&Yahr, mean ± SD	1.97 ± 0.74	2.00 ± 0.70	0.78
MDS-UPDRS III total score, mean ± SD	35.06 ± 13.85	34.53 ± 13.54	0.86
MDS-UPDRS III tremor score, mean ± SD	4.09 ± 3.65	4.88 ± 3.04	0.098
MDS-UPDRS III bradykinesia-rigidity score, mean ± SD	20.34 ± 9.68	18.38 ± 9.24	0.23
NMSS total score, mean ± SD	50.47 ± 32.73	36.83 ± 30.34	0.035
Dream enactment (possible RBD), N(%)	11 (34.4)	13 (22.4)	0.38
Rome III constipation score, mean ± SD	9.16 ± 5.61	4.24 ± 3.94	<0.001
Wexner total score, mean ± SD	7.77 ± 4.57	3.62 ± 2.91	<0.001
CSI total score, mean ± SD	20.74 ± 11.09	12.09 ± 10.25	<0.001
NMSS constipation score, mean ± SD	2.78 ± 4.22	1.03 ± 2.55	0.009

No significant differences between the groups in age, sex, MMSE, and MDS-UPDRS part III scores. Wexner and CSI are constipation questionnaires. Rome III is assessing functional gastrointestinal disorders. Abbreviations: FGID= functional gastrointestinal syndrome, N= number of patients, SD= standard deviation, MMSE= Mini-Mental State Examination, MDS-UPDRS III= MDS Unified Parkinson´s Disease Rating Scale part III, NMSS= Non-Motor Symptoms Scale, CSI= Constipation Severity Instrument
