# Supplementary material for: Gastrointestinal Symptoms and Dopamine Transporter Asymmetry in Early Parkinson's Disease
Source: Mov Disord. 2022 Mar 11;37(6):1284–9. doi: 10.1002/mds.28986 (PMC9314058; doi:10.1002/mds.28986)
Supplement: Supplementary file 4 — Table S2. PD patients divided into groups based on the mean putamen asymmetry index. [file MDS-37-1284-s001.pdf]

Table S2 PD patients divided into groups based on the mean putamen asymmetry index.

	Mean putamen imaging deficiency			p value
	right (N=32)	symmetric (N=18)	left (N=40)	
Patients, N(%)	32 (35.6)	18 (20.0)	40 (44.4)	
Age, mean ± SD	62.75 ± 11.01	68.78 ± 9.54	65.75 ± 8.92	0.11
Sex; Male, N(%)	18 (56.3)	11 (61.1)	17 (42.5)	0.33
MMSE, mean ± SD	27.66 ± 1.77	28.00 ± 1.57	27.18 ± 2.02	0.34
Motor symptoms in months, mean ± SD	31.25 ± 28.24	34.06 ± 47.69	24.75 ± 23.37	0.50
Hoehn&Yahr, mean ± SD	1.91 ± 0.59	2.17 ± 0.51	1.98 ± 0.86	0.37
MDS-UPDRS III total score, mean ± SD	32.91 ± 13.22	39.44 ± 13.34	34.05 ± 13.80	0.24
MDS-UPDRS III tremor score, mean ± SD	4.25 ± 2.95	5.33 ± 3.85	4.55 ± 3.27	0.70
MDS-UPDRS III bradykinesia-rigidity score, mean ± SD	18.84 ± 8.72	21.72 ± 9.30	18.08 ± 9.94	0.38
NMSS total score, mean ± SD	44.59 ± 33.08	31.89 ± 20.82	43.75 ± 34.37	0.43
Dream enactment (possible RBD), N(%)	10 (31.3)	6 (33.3)	8 (20.0)	0.60
Functional gastrointestinal disorders, N(%)	7 (21.9)	4 (22.2)	21 (52.5)	0.011
IBS, N(%)	4 (12.5)	1 (5.6)	12 (30.0)	0.046
Functional dyspepsia, N(%)	3 (9.4)	4 (22.2)	7 (17.5)	0.44
Rome III constipation subscore, mean ± SD	4.69 ± 4.84	5.28 ± 2.97	7.31 ± 5.87	0.13
Wexner sum, mean ± SD (N=88)	3.97 ± 3.18	3.68 ± 2.46	6.58 ± 4.80	0.044
CSI total score, mean ± SD (N=89)	12.93 ± 11.48	15.47 ± 9.33	16.94 ± 11.90	0.25
NMSS constipation score, mean ± SD	0.75 ± 2.06	1.83 ± 3.45	2.30 ± 3.94	0.18

Wexner and CSI are constipation questionnaires. Rome III is assessing functional gastrointestinal disorders. Abbreviations: N= number of patients, SD= standard deviation, MMSE= Mini-Mental State Examination, MDS-UPDRS III= MDS Unified Parkinson´s Disease Rating Scale part III, NMSS= Non-Motor Symptoms Scale, CSI= Constipation Severity Instrument
