# Supplementary material for: Gastrointestinal Symptoms and Dopamine Transporter Asymmetry in Early Parkinson's Disease
Source: Mov Disord. 2022 Mar 11;37(6):1284–9. doi: 10.1002/mds.28986 (PMC9314058; doi:10.1002/mds.28986)
Supplement: Supplementary file 5 — Table S3. SBR valuesand Z‐scorein PD patients in putamen and nucleus caudatus. [file MDS-37-1284-s005.pdf]

Table S3 SBR values and Z-score in PD patients in putamen and nucleus caudatus

Study Subject	Right Caudate		Left Caudate		Right Ant. Putamen		Left Ant. Putamen		Right Post. Putamen		Left Post. Putamen	
	SBR	Z-score	SBR	Z-score	SBR	Z-score	SBR	Z-score	SBR	Z-score	SBR	Z-score
H0018	2.16	-2	1.77	-2.9	1.79	-2.38	1.59	-2.92	1.41	-2.91	1.23	-3.02
H0020	2.51	-1.59	2.14	-2.44	2.24	-1.82	1.13	-4.09	0.82	-4.43	0.34	-5.11
H0022	2.38	-2.16	2.56	-1.95	1.57	-3.33	2.27	-2.19	0.68	-5.07	0.98	-4.16
H0024	2.57	-1.43	2.07	-2.53	2.34	-1.59	1.8	-2.73	1.32	-3.34	1	-3.72
H0028	2.4	-1.91	2.01	-2.8	2.49	-1.48	1.53	-3.42	1.29	-3.57	0.92	-4.05
H0034	2.11	-2.04	2.1	-2.21	1.78	-2.35	1.54	-2.96	1.02	-3.68	0.92	-3.61
H0047	2.79	-1.24	3.43	-0.17	1.96	-2.5	2.72	-1.18	1.13	-3.98	1.83	-2.29
H0050	2.52	-1.12	2.1	-2.08	1.95	-1.93	1.84	-2.25	1.31	-2.93	0.97	-3.36
H0051	1.82	-2.64	1.78	-2.88	1.35	-3.18	0.86	-4.34	1.24	-3.27	1.1	-3.29
H0059	2.12	-1.81	1.89	-2.4	1.48	-2.69	1.81	-2.22	1.04	-3.4	0.75	-3.72
H0062	2.48	-1.36	2.13	-2.18	2.37	-1.31	1.85	-2.39	1.67	-2.35	1.07	-3.32
J0001	1.71	-3.04	1.93	-2.77	1.23	-3.57	1.38	-3.51	1.21	-3.53	1.16	-3.37
J0002	2.51	1.25	2.54	-1.33	1.84	-2.22	1.82	-2.39	1.38	-2.9	1.45	-2.5
J0006	3.94	1.05	3.38	-0.14	3.26	-0.06	2.57	-1.36	2.77	-0.47	1.21	-3.44
J0007	2.94	-1.36	3.86	0.27	2.48	-1.93	2.87	-1.3	0.98	-4.73	1.93	-2.51
J0013	3.15	0.12	2.8	-0.68	2.61	-0.68	2.32	-1.25	1.82	-1.82	1.06	-3.12
J0023	3.27	-0.48	3.64	0.09	2.16	-2.3	2.88	-1.03	1.07	-4.28	1.56	-3.01
J0049	2.9	-0.42	2.33	-1.66	2.76	-0.49	2	-1.96	1.89	-1.75	0.88	-3.56
J0050	2.22	-2.04	2.5	-1.66	1.82	-2.48	1.74	-2.79	0.71	-4.54	0.81	-4.05
J0053	2.74	-0.74	2.66	-1.04	2.19	-1.51	1.86	-2.24	1.01	-3.57	0.64	-4.06
J0055	3.24	-0.22	3.08	-0.67	2.44	-1.49	1.92	-2.57	1.4	-3.26	1.07	-3.67
J0057	2.48	-1.07	2.32	-1.52	2.02	-1.67	1.74	-2.31	1.26	-2.89	1.02	-3.11
J0063	1.49	-3.34	1.58	-3.32	1.19	-3.52	1.12	-3.89	0.62	-4.6	0.91	-3.72
J0066	2.25	-1.62	2.14	-1.98	1.83	-2.11	1.63	-2.63	1.51	-2.49	1.28	-2.7

Table S3 SBR values and Z-score in PD patients in putamen and nucleus caudatus (continued)

Study Subject	Right Caudate		Left Caudate		Right Ant. Putamen		Left Ant. Putamen		Right Post. Putamen		Left Post. Putamen	
	SBR	Z-score	SBR	Z-score	SBR	Z-score	SBR	Z-score	SBR	Z-score	SBR	Z-score
J0068	3.02	-0.46	2.96	-0.72	2.97	-0.37	2.07	-2.1	2.21	-1.38	0.68	-4.26
J0070	1.88	-2.66	1.95	-2.69	1.19	-3.6	1.35	-3.52	0.99	-3.94	0.89	-3.85
J0076	2.69	-0.96	3.05	-0.41	1.87	-2.22	2.26	-1.58	0.87	-4	0.97	-3.52
J0077	4.81	2.69	4.33	1.65	4.82	2.73	2.88	-0.79	4.33	2.71	0.89	-4.12
J0087	2.23	-1.66	2.64	-1.01	2.15	-1.54	2.35	-1.23	1.41	-2.69	1.5	-2.25
J0089	2.46	-1.48	2.7	-1.15	2.06	-1.95	2.06	-2.06	1.07	-3.67	1.37	-2.8
J0092	2.24	-2.01	1.64	-3.3	1.93	-2.28	1.43	-3.39	1.43	-3.05	1.04	-3.58
J0099	1.46	-3.51	1.94	-2.75	1.09	-3.83	1.32	-3.64	0.82	-4.34	0.93	-3.84
J0102	2.25	-2.35	3.62	0.12	1.7	-3.05	2.98	-0.76	1.28	-3.76	2.5	-1.01
J0103	2.54	-1.49	3.07	-0.61	1.52	-3.08	2.81	-0.76	0.63	-4.75	1.86	-1.96
J0104	1.94	-2.4	1.97	-2.48	1.11	-3.59	1.54	-2.98	0.95	-3.84	1.05	-3.36
J0107	1.73	-2.71	1.91	-2.52	1.24	-3.28	1.3	-3.38	0.84	-3.98	1.03	-3.32
J0109	2.51	-1.75	2.69	-1.55	2.06	-2.29	1.85	-2.85	0.78	-4.68	0.77	-4.41
J0113	2.65	-0.93	2.91	-0.57	2.17	-1.58	2.27	-1.46	1.32	-2.97	1.74	-1.85
J0114	2.78	-0.92	2.37	-1.86	2.47	-1.26	2.11	-2.01	1.84	-2.14	1.39	-2.81
J0117	2.49	-1.79	2.12	-2.66	1.82	-2.73	1.25	-4.02	0.99	-4.25	0.56	-4.84
T0057	2.16	-2.1	2.51	-1.57	1.58	-2.87	2.01	-2.21	0.79	-4.32	0.67	-4.28
T0061	1.68	-3.33	2.46	-1.97	1.18	-3.86	1.57	-3.36	0.83	-4.55	0.89	-4.15
T0062	3.01	-0.08	3.03	-0.18	2.23	-1.31	2.07	-1.67	1.18	-3.08	1.08	-3.02
T0063	1.61	-2.92	1.86	-2.6	1.1	-3.52	1.56	-2.85	0.63	-4.4	0.81	-3.75
T0064	1.71	-2.64	1.92	-2.39	1.03	-3.55	1.14	-3.57	0.51	-4.54	0.71	-3.85
T0065	1.87	-2.39	2.4	-1.52	1.2	-3.3	1.91	-2.14	1.05	-3.49	0.96	-3.39
T0074	2.12	-2.49	2.84	-1.26	1.32	-3.62	1.95	-2.63	0.9	-4.42	0.87	-4.21
T0075	1.9	-3.13	2.33	-2.46	1.08	-4.26	1.3	-4.13	0.76	-4.96	1.27	-3.61
T0077	1.43	-3.1	1.24	-3.62	0.89	-3.74	0.84	-4.09	0.67	-4.15	0.6	-3.99

Table S3 SBR values and Z-score in PD patients in putamen and nucleus caudatus (continued)

Study Subject	Right Caudate		Left Caudate		Right Ant. Putamen		Left Ant. Putamen		Right Post. Putamen		Left Post. Putamen	
	SBR	Z-score	SBR	Z-score	SBR	Z-score	SBR	Z-score	SBR	Z-score	SBR	Z-score
T0080	1.91	-2.52	1.6	-3.26	1.26	-3.39	1.03	-4.06	0.85	-4.12	0.64	-4.28
T0089	1.79	-2.72	1.55	-3.33	1.72	-2.52	1.1	-3.88	1.02	-3.74	0.71	-4.1
T0090	2.92	-1.04	2.33	-2.31	2.28	-1.97	2.07	-2.49	1.06	-4.18	0.72	-4.59
T0098	1.26	-3.48	1.75	-2.7	0.87	-3.84	1.07	-3.71	0.59	-4.37	0.81	-3.65
T0099	0.87	-4.38	0.96	-4.38	0.98	-3.78	0.76	-4.46	0.8	-4.11	0.67	-4.1
T0101	1.26	-3.51	1.35	-3.48	0.98	-3.64	0.68	-4.47	0.62	-4.32	0.38	-4.53
T0107	1.39	-3.2	1.91	-2.36	1.48	-2.69	1.37	-3.07	0.89	-3.71	0.87	-3.48
T0123	1.98	-2.38	1.94	-2.61	1.8	-2.42	1.48	-3.18	0.79	-4.26	0.46	-4.64
T0124	2.2	-2.1	1.8	-3.01	1.54	-3	1.39	-3.48	0.91	-4.14	0.38	-4.94
T0125	2.56	-1.83	2.36	-2.36	2.12	-2.36	1.42	-3.85	1.32	-3.75	0.58	-4.97
T0132	2.17	-2.19	2.48	-1.73	1.31	-3.45	1.64	-3.03	0.62	-4.79	0.99	-3.74
T0147	1.97	-2.3	1.94	-2.51	1.87	-2.19	0.97	-4.07	1.55	-2.57	0.58	-4.29
T0150	2.1	-2.32	2.23	-2.22	1.59	-2.95	1.94	-2.44	1.48	-3	1.46	-2.78
T0163	1.23	-3.5	1.21	-3.71	0.74	-4.02	1.12	-3.56	0.93	-3.61	0.65	-3.92
T0173	1.72	-2.84	1.91	-2.62	1.19	-3.47	1.28	-3.52	0.52	-4.75	0.68	-4.15
T0177	1.94	-2.37	2.91	-0.66	1.37	-3.1	2.25	-1.59	0.93	-3.85	1.65	-2.11
T0184	1.53	-3.18	1.88	-2.66	0.49	-4.72	1.43	-3.21	0.46	-4.86	0.63	-4.22
T0188	1.72	-2.8	2.06	-2.29	1.52	-2.83	1.91	-2.25	1.25	-3.2	1.18	-3.08
T0191	1.68	-2.99	1.8	-2.9	1.29	-3.36	1.03	-4.07	0.76	-4.35	0.52	-4.55
T0192	1.75	-2.69	1.79	-2.76	1.69	-2.47	1.75	-2.5	1.38	-2.88	1.34	-2.69
T0196	2.18	-2.04	1.62	-3.27	2.28	-1.59	1.25	-3.66	1.54	-2.73	0.6	-4.39
T0197	1.52	-3.09	0.78	-4.66	1.24	-3.24	0.61	-4.68	1.19	-3.22	0.5	-4.36
T0199	2.15	-1.95	1.83	-2.71	1.68	-2.51	1.33	-3.35	0.83	-4.04	0.75	-3.93

Table S3 SBR values and Z-score in PD patients in putamen and nucleus caudatus (continued)

Study Subject	Right Caudate		Left Caudate		Right Ant. Putamen		Left Ant. Putamen		Right Post. Putamen		Left Post. Putamen	
	SBR	Z-score	SBR	Z-score	SBR	Z-score	SBR	Z-score	SBR	Z-score	SBR	Z-score
T0201	1.98	-2.01	1.86	-2.39	1.25	-3.06	1.16	-3.43	0.79	-3.84	0.52	-4.12
T0203	2.97	-0.96	2.44	-2.12	2.66	-1.28	1.98	-2.68	1.97	-2.29	0.97	-4.08
T0211	1.44	-3.16	1.4	-3.38	0.76	-4.05	1.26	-3.33	0.54	-4.49	0.93	-3.4
T0215	0.78	-4.94	1.01	-4.66	0.65	-4.73	0.93	-4.51	0.67	-4.78	0.37	-5.1
T0219	1.43	-3.12	1.82	-2.52	1.11	-3.35	1.75	-2.32	0.68	-4.14	1.21	-2.78
T0224	1.51	-3.27	1.41	-3.61	1.67	-2.63	1.1	-3.9	0.97	-3.87	0.47	-4.61
T0225	1.37	-3.35	1.72	-2.84	1.06	-3.55	1.15	-3.61	0.6	-4.42	0.39	-4.57
T0227	1.92	-2.25	1.78	-2.66	1.35	-2.99	1.14	-3.58	0.96	-3.61	0.76	-3.75
T0233	2.93	-0.65	2.78	-1.09	1.92	-2.28	1.45	-3.32	1.08	-3.75	0.89	-3.85
T0237	1.79	-2.72	2.59	-1.33	1.52	-2.88	1.79	-2.55	0.52	-4.78	1.39	-2.71
T0238	2.24	-2.24	2.6	-1.69	1.77	-2.8	1.93	-2.66	1.1	-4	1.14	-3.63
T0242	2.49	-1.53	2.51	-1.64	2.02	-2.12	2.11	-2.07	1.64	-2.61	1.81	-2.02
T0246	1.61	-3.09	1.25	-3.92	1.13	-3.61	1.24	-3.64	0.41	-5.03	0.59	-4.36
T0247	1.63	-3.06	1.58	-3.31	0.96	-3.93	1.2	-3.73	0.29	-5.3	0.71	-4.13
T0250	2	-2.64	2.16	-2.49	1.44	-3.35	1.56	-3.32	0.73	-4.7	1.08	-3.7
T0252	2.01	-1.98	1.86	-2.41	1.42	-2.76	1.22	-3.31	0.73	-3.98	0.93	-3.3
T0259	1.5	-3.13	1.57	-3.14	1.28	-3.18	1.09	-3.76	0.65	-4.36	0.46	-4.46
T0261	1.64	-2.91	1.67	-3.01	1.38	-3.05	1.2	-3.6	1	-3.68	0.73	-3.97

Abbreviations: SBR= spesific binding ratio, Z-score= Age-adjusted standard score; standard deviations from the mean
