# Supplementary material for: Gastrointestinal Symptoms and Dopamine Transporter Asymmetry in Early Parkinson's Disease
Source: Mov Disord. 2022 Mar 11;37(6):1284–9. doi: 10.1002/mds.28986 (PMC9314058; doi:10.1002/mds.28986)
Supplement: Supplementary file 6 — Appendix S1. Supporting Information. [file MDS-37-1284-s003.pdf]

SUPPLEMENTARY MATERIAL

MATERIALS AND METHODS

In this cross-sectional clinical and imaging study Inclusion and exclusion criteria were:

The study subjects were required to be aged 18 or over and to be able to understand and answer the questionnaires in Finnish. Subjects with any limitation affecting their ability to understand the informed consent, such as significant mental health problems or cognitive problems (Mini-Mental State Examination (MMSE) < 18), were excluded.

The scanning was performed for 399 patients. Of the scanned patients, 248 returned the questionnaires regarding GI symptoms. Of these, 90 patients were eventually diagnosed with PD (clinical evaluation after the imaging, based on abnormal DAT scan and clinical symptoms of PD).

SPECT imaging and data analysis

SPECT imaging was carried out with different systems (and collimators) in different sites (Siemens Symbia T6 (low-energy high-resolution, (LEHR)), Phillips Brightview XCT (LEHR) two GE Infinia II Hawkeyes (LEHRs), Siemens Intevo (Fanbeam), GE NM/CT 670 ES (LEHR)) and the results were scanner-specific corrected and patient-age corrected for the specific binding ratio (SBR).[1] The image quality was inspected visually by the investigators before and after image processing.

The classification of patients to normal and abnormal DAT binding groups were based on the automated semi-quantitative BRASS-analysis. Scans with borderline results were re-evaluated by a movement disorder specialist with extensive experience in brain dopamine imaging (VK).

AI in DAT uptake was also calculated for posterior putamen and nucleus caudatus the same way as for mean putamen. AI was also calculated so that left-predominant, symmetric and right-predominant reduction of DAT binding could be differentiated and these were used for further analyses done with these three groups. For absolute values see Table S3.

Clinical features

MDS-UPDRS tremor scores included items for postural and kinetic tremor of the hand, upper and lower extremity rest tremor. Bradykinesia and rigidity scores included items for upper and lower extremity rigidity, finger and toe tapping, hand movements, pronation-supination movements of the hand, and leg agility. The AI for the parkinsonian features was calculated: AI= (MDS-UPDRS highest – MDS-UPDRS lowest)/(MDS-UPDRS highest + MDS-UPDRS lowest).

In CSI, Wexner and Rome III questionnaires if there were more than 20% of answers missing the value was labeled missing. If there were less than 20% of answers missing, the values were corrected for the missing ones (value*questions/(questions-unanswered questions)[2].

Imaging asymmetry and clinical symptoms

We aimed to find out the best nucleus to be used in laterality analyses. The motor symptoms AI correlated with the imaging AI (Pearson correlation coefficient 0.69 ($p<0.001$) for mean putamen,0.64 ($p<0.001$) for posterior putamen and 0.61($p<0.001$) for nucleus caudatus). Therefore, the mean putamen SBR was used for the analyses. Imaging asymmetry correlated with observed contralateral motor symptoms.

Statistical analyses

For continuous variables, we evaluated the assumption of normality visually from histograms together with Kolmogorov-Smirnov and Shapiro-Wilk tests. Independent sample T-test, Mann-Whitney U and Chi-Square tests were used to investigate group differences in the continuous and categorical variables. One-Way ANOVA and Kruskal-Wallis test were used to investigate differences in total Wexner score, Rome III total score and total CSI score and its subscales with respect to asymmetry index groups (right-predominant reduction of DAT binding, symmetrically reduced DAT binding and left-predominant reduction of DAT binding). P values less than 0.05 were considered significant. Since this was an explorative study into potential connections between DAT asymmetry and clinical symptoms, results were not adjusted for multiple comparisons[3].

References

1. Albert NL, Unterrainer M, Diemling M, et al. Implementation of the european multicentre database of healthy controls for [(123)I]FP-CIT SPECT increases diagnostic accuracy in patients with clinically uncertain parkinsonian syndromes. Eur J Nucl Med Mol Imaging 2016; 43: 1315-1322.

2. Joutsa J, Martikainen K, Vahlberg T, Voon V, Kaasinen V. Impulse control disorders and depression in finnish patients with parkinson's disease. Parkinsonism Relat Disord 2012; 18: 155-160.

3. Bender R, Lange S. Adjusting for multiple testing—when and how? J Clin Epidemiol 2001; 54(4): 343-349.
